# Predictors of recurrence free survival for patients with stage II and III colon cancer

**DOI:** 10.1186/1471-2407-14-336

**Published:** 2014-05-16

**Authors:** Vassiliki L Tsikitis, David W Larson, Marianne Huebner, Christine M Lohse, Patricia A Thompson

**Affiliations:** 1 Department of Surgery, Oregon Health & Science University, Portland, Oregon, USA; 2 Department of Surgery, The Mayo Clinic, Rochester, Minnesota, 200 First Street SW, 55905 Rochester, MN, USA; 3 Department of Health Sciences Research, The Mayo Clinic, Rochester, Minnesota, 200 First Street SW, 55905 Rochester, MN, USA; 4 Department of Cellular and Molecular Medicine, University of Arizona, Tucson, Arizona, 1333 N. Martin Avenue, 85721 Tucson, Arizona, USA

**Keywords:** Chemotherapy, Disease-free survival, Early stage colon cancer, Clinico-pathologic, Predictors of recurrence

## Abstract

**Background:**

The aim of this study was to evaluate clinico-pathologic specific predictors of recurrence for stage II/III disease. Improving recurrence prediction for resected stage II/III colon cancer patients could alter surveillance strategies, providing opportunities for more informed use of chemotherapy for high risk individuals.

**Methods:**

871 stage II and 265 stage III patients with colon cancers were included. Features studied included surgery date, age, gender, chemotherapy, tumor location, number of positive lymph nodes, tumor differentiation, and lymphovascular and perineural invasion. Time to recurrence was evaluated, using Cox’s proportional hazards models. The predictive ability of the multivariable models was evaluated using the concordance (*c*) index.

**Results:**

For stage II cancer patients, estimated recurrence-free survival rates at one, three, five, and seven years following surgery were 98%, 92%, 90%, and 89%. Only T stage was significantly associated with recurrence. Estimated recurrence-free survival rates for stage III patients at one, three, five, and seven years following surgery were 94%, 78%, 70%, and 66%. Higher recurrence rates were seen in patients who didn’t receive chemotherapy (p = 0.023), with a higher number of positive nodes (p < 0.001). The *c*-index for the stage II model was 0.55 and 0.68 for stage III.

**Conclusions:**

Current clinic-pathologic information is inadequate for prediction of colon cancer recurrence after resection for stage II and IIII patients. Identification and clinical use of molecular markers to identify the earlier stage II and III colon cancer patients at elevated risk of recurrence are needed to improve prognostication of early stage colon cancers.

## Background

Colorectal cancer represents the most commonly diagnosed gastrointestinal cancer and the third most common cause of cancer-related death in the United States [1]. The current TNM staging system for colorectal cancer is based on three elements: the penetration of tumor into the intestinal wall (T), the number of positive lymph nodes present (N), and the presence of metastasis (M). For patients without metastatic disease, surgery offers the only curative option. Chemotherapy is largely reserved for patients with positive lymph nodes (stage III disease) [2], because it can reduce the risk of disease recurrence by 40 to 50%.

Clinicians do not currently question the benefit of chemotherapy for stage III colon cancer patients, despite the fact that 50% of these patients will eventually develop metastatic disease. Results of the Quick and Simple and Reliable (QUASAR) study implied that certain patients with stage II colon cancer (T3, T4/N0) may have more favorable outcomes with adjuvant therapy [3]. Despite being controversial, chemotherapy for stage II disease is advised for patients with poor prognostic factors including T4 stage, less than 12 lymph nodes sampled at the time of resection, clinical bowel obstruction and perforation, and poor histologic grade with lymphovascular and perineural invasion [3,4]. The predictive accuracy of those clinico-pathologic characteristics has not been evaluated independently for stages II and III colon cancer. In this study, we aimed to examine the performance of those clinical predictors of recurrence-free survival for stage II and III colon cancer patients who were treated in our institution.

## Methods

### Patient selection

Eight hundred seventy-one patients with stage II colon cancer treated surgically between 1995 and 2007 and 265 patients with stage III colon cancer treated surgically between 1996 and 2001 were available for study. All patients had signed consent to be included in the study, and the appropriate approval from the Mayo Clinic Institutional Review Board (IRB) had been obtained.

### Clinical and pathologic features

The clinical and pathologic features studied for patients with stage II colon cancer included year of surgery, age at surgery, sex, adjuvant chemotherapy, tumor location, primary tumor size, primary tumor classification, the total number of lymph nodes examined, and tumor differentiation. The clinical and pathologic features studied for patients with stage III colon cancer included year of surgery, age at surgery, sex, adjuvant chemotherapy, tumor location, primary tumor size, primary tumor classification, regional lymph node involvement, the numbers of positive, negative, and total lymph nodes; tumor differentiation, lymphovascular invasion, and perineural invasion.

### Follow-up and recurrence of disease

We included all recurrences in this patient population, both local (anastomotic and regional) and distant (hepatic and lung metastases). The follow-up included a colonoscopy one year after surgery, with a yearly CT of the abdomen and pelvis every year for stage II and III disease, for up to five years. The frequency of repeat colonoscopies depended on the findings of the first surveillance colonoscopy. Patients with normal exam had a repeat colonoscopy three years later. Chest examination consisted of a chest x-ray, though current NCCN guidelines call for a chest CT. The patients were primarily followed by either their medical oncologists or a colorectal surgeon within our institution. As our institution is a large tertiary referral center, a high number of patients elected to be surveyed by local physicians. Those patients have been excluded due to lack of data for follow-up. In our study, we have included only patients who had recurrences either reported at their six-month surveillance visit or at a later date. The follow-up period for this cohort of patients by our institutions’ oncology team ranged up to ten years. There is a comprehensive multidisciplinary approach for all cancer patients and surveillance after colon cancer surgery is primarily carried by our medical oncologists. The recurrences we reported are not second primaries; these patients are followed closely, as our institution is part of the National Cancer Database sites.

### Statistical methods

Continuous features were summarized with means, standard deviations (SD), medians, and ranges. Categorical features were summarized with frequency counts and percentages. Changes in features by year of surgery were evaluated, using Spearman rank correlation coefficients, Kruskal-Wallis and Wilcoxon rank sum tests, and chi-square tests. Recurrence-free survival rates were estimated, using the Kaplan-Meier method. Associations of the features studied with time to recurrence were evaluated, using Cox proportional hazards regression models and summarized with hazard ratios and 95% confidence intervals (CIs). Multivariable models were developed, using stepwise selection with a significance level for a feature to enter or leave the model of 0.05. The predictive ability of the features in a model was evaluated, using the *c* (for concordance) index proposed by Harrell et al. [5]. The interpretation of the c-index is identical to the interpretation of the area under a receiver operating characteristic curve. A *c*-index of 1.0 indicates that the features in the model perfectly separate patients with different outcomes, while a value of 0.5 indicates that the features contain prognostic information equal to that obtained by chance alone. Statistical analyses were performed, using the SAS software package (SAS Institute, Cary, NC). All tests were two-sided and p-values <0.05 were considered statistically significant.

## Results

### Stage II

Clinical and pathologic features for the 871 patients with stage II colon cancer are summarized in Table 1. The total number of lymph nodes retrieved and examined was significantly and positively correlated with year of surgery (Spearman rank correlation coefficient 0.37; p < 0.001). For example, the mean number of total lymph nodes for patients treated between 1995 and 2001 was 14.1, which increased to 20.5 for patients treated between 2002 and 2007 (p < 0.001). The distribution of tumor differentiation also changed significantly over time. There were 42%, 50%, and 8% well, moderately, and poorly differentiated tumors among patients treated between 1995 and 2001, compared with 21%, 72%, and 7% well, moderately, and poorly differentiated tumors among patients treated between 2002 and 2007 (p < 0.001).

**Table 1:** Summary of clinical and pathologic features for 871 patients with stage II colon cancer

**Patient/Tumor characteristics**	
Age at surgery (years, mean ± SD)	71.1 ± 12.0
Primary tumor size (mm; mean ± SD)^†^	51.9 ± 22.8
Total number lymph nodes^†^	16.5 ± 10.0
Sex (N,%)	
Female	440 (51)
Male	431 (49)
Adjuvant chemotherapy (N,%)^†^	
No	677 (80)
Yes	170 (20)
Tumor location (N,%)	
Right	510 (59)
Sigmoid	192 (22)
Transverse	100 (11)
Left	69 (8)
Primary tumor classification (N,%)	
T3	813 (93)
T4	58 (7)
Tumor differentiation (N,%)^†^	
Well	315 (33)
Moderate	566 (60)
Poor	70 (7)

^†^Sample size for tumor size (N = 868), for total lymph nodes (N = 870), for adjuvant chemotherapy (N = 847) and for tumor differentiation (N = 869).

At last follow-up, 87 patients experienced recurrence at a mean of 2.3 years following surgery (median 1.9 years). Among the 857 patients who did not experience a recurrence, the mean duration of follow-up was 7.0 years (median 6.5 years). Estimated recurrence-free survival rates (95% CI; number still at risk) at one, three, five, seven, and ten years following surgery were 98% (97 – 99; 822), 92% (90 – 94; 674), 90% (88 – 92; 512), 89% (87 – 91; 371), and 89% (86 – 91; 195), respectively. Univariate associations of the clinical and pathologic features studied with recurrence are summarized in Table 2. Only primary stage classification was significantly associated with recurrence. Patients with T4 tumors were over three times more likely to recur, compared with patients with T3 tumors (hazard ratio (HR) 3.17; p < 0.001). The *c*-index from this univariate model was 0.55. Estimated recurrence-free survival rates by primary tumor classification are summarized in Table 3. Of note, after adjusting for T stage (T4 versus T3), no other feature, including chemotherapy, was statistically associated with time to recurrence. Analyzing the data from the total of 58 T4 patients in our cohort, six had missing data regarding their chemotherapy; of the remaining 52, 26 (50%) were treated with % FU based chemotherapy. In this subset, chemotherapy was not statistically associated with time to recurrence (HR2.48; 95% CI 0.78-7.91; p = 0.12).

**Table 2:** Univariate associations of clinical and pathologic features with recurrence for 871 patients with stage II colon cancer

**Patient/Tumor characteristics**	**Hazard ratio (95% CI)**	**P-value**
Year of surgery (1-year increase)	0.97 (0.92 – 1.04)	0.38
Age at surgery (10-year increase)	0.91 (0.76 – 1.08)	0.26
Sex		
Female	1.0 (reference)	
Male	1.20 (0.79 – 1.83)	0.40
Adjuvant chemotherapy^†^		
No	1.0 (reference)	
Yes	1.41 (0.87 – 2.27)	0.16
Tumor location		
Right	1.0 (reference)	
Sigmoid	1.37 (0.85 – 2.19)	0.20
Transverse	0.69 (0.32 – 1.53)	0.37
Left	0.58 (0.21 – 1.61)	0.30
Tumor location		
Right, transverse, or left	1.0 (reference)	
Sigmoid	1.50 (0.95 – 2.37)	0.08
Primary tumor size (20-mm increase)^†^	0.96 (0.80 – 1.15)	0.66
Primary tumor classification		
T3	1.0 (reference)	
T4	3.17 (1.79 – 5.61)	<0.001
Total lymph nodes (10-node increase)^†^	0.84 (0.65 – 1.07)	0.15
Tumor differentiation^†^		
Well	1.0 (reference)	
Moderate	1.20 (0.76 – 1.89)	0.45
Poor	1.07 (0.44 – 2.59)	0.88
Tumor differentiation^†^		
Well or moderate	1.0 (reference)	
Poor	0.96 (0.42 – 2.20)	0.92

^†^Sample size for tumor size (N = 868), for total lymph nodes (N = 870), for adjuvant chemotherapy (N = 847) and for tumor differentiation (N = 869).

**Table 3:** Estimated recurrence-free survival rates (95% CI; number still at risk) by primary tumor classification for 871 patients with stage II colon cancer

**Year**	**T3 (N = 813)**	**T4 (N = 58)**
1	98% (97 – 99; 771)	95% (89 – 100; 51)
3	93% (91 – 95; 638)	79% (68 – 91; 36)
5	91% (89 – 93; 489)	72% (60 – 86; 23)
7	90% (88 – 92; 352)	72% (60 – 86; 19)
10	90% (87 – 92; 184)	72% (60 – 86; 11)

### Stage III

Clinical and pathologic features for the 265 patients with stage III colon cancer are summarized in Table 4. The number of negative lymph nodes and the total number of lymph nodes examined were significantly and positively correlated with year of surgery (Spearman rank correlation coefficients of 0.22 and 0.22). None of the other features studied changed significantly over time.

**Table 4:** Summary of clinical and pathologic features for 265 patients with stage III

**Patient/Tumor characteristic**	
Age at surgery (years, mean ± SD)	68.3 ± 12.5
Primary tumor size (mm; mean ± SD)^†^	47.2 ± 21.7
Positive lymph nodes (mean ± SD	2.3 ± 3.0
Negative lymph nodes (mean ± SD)	12.1 ± 8.3
Total lymph nodes (mean ± SD)	14.9 ± 8.8
Sex	**N (%)**
Female	134 (51)
Male	131 (49)
Adjuvant chemotherapy	
No	73 (28)
Yes	192 (72)
Tumor location^†^	
Right	123 (47)
Sigmoid	77 (30)
Transverse	31 (12)
Left	28 (11)
Primary tumor classification^†^	
T1	12 (5)
T2	27 (10)
T3	205 (78)
T4	19 (7)
Regional lymph node involvement	
N1	203 (77)
N2	62 (23)
Tumor differentiation^†^	
Well	78 (30)
Moderate	161 (61)
Poor	25 (9)
Lymphovascular invasion	
No	256 (97)
Yes	9 (3)
Perineural invasion	
No	262 (99)
Yes	3 (1)

^†^Sample size for tumor size (N = 263), for tumor location (N = 259), for primary tumor classification (N = 263) and for tumor differentiation (N = 264).

At last follow-up, 79 patients experienced recurrence at a mean of 2.5 years following surgery (median 1.8 years). Among the 186 patients who did not experience a recurrence, the mean duration of follow-up was 5.4 years (median 5.1 years). Estimated recurrence-free survival rates (95% CI; number still at risk) at one, three, five, and seven years following surgery were 94% (91 – 97; 243), 78% (73 – 83; 187), 70% (65 – 77; 106), and 66% (59 – 73; 55), respectively. Univariate associations of the clinical and pathologic features studied with recurrence are summarized in Table 5. The multivariable model developed, using these features, is summarized in Table 6. Patients treated with adjuvant chemotherapy were significantly less likely to recur, compared with those who were not treated (HR = 0.57; (0.35 – 0.93 95% CI) p < 0.023). After adjusting for adjuvant chemotherapy, each one-node increase in the number of positive lymph nodes was associated with a 24% increased risk of recurrence (HR = 1.24; (1.18 – 1.31 95% CI) p < 0.001). Markedly, even after adjusting for the total lymph nodes, which we recognize differed across patients, an increase in the number of positive lymph nodes is still significantly associated with time to recurrence. The *c*-index from this multivariable model was 0.68. Estimated recurrence-free survival rates by primary tumor classification are summarized in Table 7.

**Table 5:** Univariate associations of clinical and pathologic features with recurrence for 265 patients with stage III colon cancer

**Patient and tumor characteristics**	**Hazard ratio (95% CI)**	**P-value**
Year of surgery (1-year increase)	0.98 (0.86 – 1.11)	0.71
Age at surgery (10-year increase)	1.10 (0.91 – 1.33)	0.31
Sex		
Female	1.0 (reference)	
Male	1.16 (0.75 – 1.80)	0.51
Adjuvant chemotherapy		
No	1.0 (reference)	
Yes	0.68 (0.42 – 1.09)	0.11
Tumor location^†^		
Right	1.0 (reference)	
Sigmoid	0.82 (0.49 – 1.38)	0.46
Transverse	1.01 (0.50 – 2.03)	0.98
Left	0.78 (0.35 – 1.75)	0.55
Tumor location^†^		
Right, transverse, or left	1.0 (reference)	
Sigmoid	0.85 (0.52 – 1.39)	0.51
Primary tumor size (20-mm increase)^†^	1.16 (0.96 – 1.41)	0.12
Primary tumor classification		
T1	1.0 (reference)	
T2	3.70 (0.46 – 30.05)	0.22
T3	4.34 (0.60 – 31.34)	0.15
T4	9.59 (1.23 – 74.94)	0.031
Primary tumor classification^†^		
T1, T2, or T3	1.0 (reference)	
T4	2.35 (1.21 – 4.57)	0.012
Regional lymph node involvement		
N1	1.0 (reference)	
N2	3.23 (2.06 – 5.06)	<0.001
Positive lymph nodes (1-node increase)	1.23 (1.17 – 1.30)	<0.001
Negative lymph nodes (10-node increase)	1.04 (0.78 – 1.37)	0.80
Total lymph nodes (10-node increase)	1.34 (1.06 – 1.69)	0.013
Tumor differentiation^†^		
Well	1.0 (reference)	
Moderate	1.50 (0.89 – 2.52)	0.13
Poor	1.90 (0.86 – 4.17)	0.11
Tumor differentiation^†^		
Well or moderate	1.0 (reference)	
Poor	1.45 (0.72 – 2.90)	0.30
Lymphovascular invasion		
No	1.0 (reference)	
Yes	2.37 (0.95 – 5.89)	0.06
Perineural invasion		
No	1.0 (reference)	
Yes	0.99 (0.14 – 7.14)	0.99

^†^Sample size for tumor size (N=263), for tumor location (N=259), for primary tumor classification (N=263) and for tumor differentiation (N=264).

**Table 6:** Multivariable model to predict recurrence for 265 patients with stage III colon cancer

**Patient/Tumor characteristic**	**Hazard Ratio (95% CI)**	**P-value**
Adjuvant chemotherapy		
No	1.0 (reference)	
Yes	0.57 (0.35 – 0.93)	0.023
Positive lymph nodes (1-node increase)	1.24 (1.18 – 1.31)	<0.001

**Table 7:** Estimated recurrence-free survival rates (95% CI; number still at risk) by primary tumor classification for 265 patients with stage III colon cancer

**Year**	**T1 (N = 12)**	**T2 (N = 27)**	**T3 (N = 205)**	**T4 (N = 19)**
1	100% (100 – 100; 12)	100% (100 – 100; 26)	94% (90 – 97; 187)	89% (76 – 100; 16)
3	100% (100 – 100; 12)	80% (65 – 97; 19)	78% (72 – 84; 144)	61% (42 – 88; 10)
5	89% (71 – 100; 6)	73% (56 – 95; 11)	70% (63 – 77; 81)	55% (36 – 84; 7)
7	89% (71 – 100; 2)	73% (56 – 95; 7)	66% (59 – 74; 43)	38% (19 – 74; 3)

## Discussion

The results of this study, analyzing the data of a total of 871 patients with stage II colon cancer, demonstrated a five-year recurrence rate of 10%. Most recurrences occurred in the first two years after surgery. The prognostic factor identified was the T stage. The population of the 265 patients with stage III colon cancer had, as expected, a much higher five-year recurrence rate of 30%, with most recurrences occurring within the first two years after surgery. The clinical prognostic factors for stage III colon cancer included the number of positive lymph nodes and the use of adjuvant chemotherapy.

Compared with other studies, the findings for risk of recurrence for stage II and III colon cancer are similar to those in our findings [6]. The 7^th^ edition of the American Joint Commission on Cancer (AJCC) [7] further classified T4 stage II tumors into the sub categories of T4a and T4b. This change was the result of observed differences in outcomes within the T4 classification, based on the tumor spread through the bowel wall either to just serosa (T4a) or to adjacent organs (T4b). The study that supported this finding examined 119,363 colon cancer patients from the Surveillance, Epidemiology, and End Results (SEER) database and showed that the survival rate of patients with stage IIB was lower than those with stage IIIA. The authors attributed this finding to the following factors: first, that patients with stage III received adjuvant treatment and therefore fared better than those with stage II disease that did not receive chemotherapy, and second, that patients with stage T4 N1 tumors might have been understaged as stage T4 N0 tumors. The first argument has been challenged by another study that had not shown statistically significant differences in survival among patients with stage IIB and IIIA disease [8]. To support the argument that patients with stage IIB disease fare worse than patients with stage IIIA, a Dutch study that examined 2,282 patients with all stages of colorectal cancer [9] demonstrated that patients with stage IIB tumors had a higher risk of developing locoregional recurrence when compared to patients with stage IIIA.

None of the other factors, including total number of lymph nodes, lymphovascular and perineural invasion, and tumor differentiation or clinical obstruction at the time of diagnosis were significantly associated with the risk of recurrence for the patients with stage II colon cancer. These findings differ from other studies demonstrating that certain pathologic characteristics, such as histologic grade, carry prognostic value. In particular, in a study of 1,031 patients who underwent a curative resection for colon adenocarcinoma, tumor differentiation was related to local recurrence with no events for patients with well-differentiated tumors. In comparison, patients with poorly differentiated tumors experienced a 6.8% risk of local, regional, or distant recurrence [6].

The 7^th^ edition of AJCC [7] emphasizes that at least 10–14 nodes should be retrieved in colon specimens for adequate staging. In our study, the mean number of lymph nodes was 16.5. Notably, the total number of lymph nodes examined was positively correlated with year of surgery (Spearman rank correlation coefficient 0.37; p < 0.001), an increase from a mean of 14.1, between years 1995 and 2001, to a mean of 20.5 for patients treated between 2002 and 2007 (p < 0.001). This increase of total number retrieval, however, did not improve disease-free survival rates for this 871 patient cohort. Studies focusing on stage II disease suggest that patients with fewer total lymph nodes retrieved at surgery fare worse than those who had a high number of total nodes recovered and examined [10,11]. This argument is founded on the potential of stage migration and the encounter of micrometastases, a finding that was not observed in our study. Furthermore, a c-index of 0.55 indicates that we cannot adequately predict recurrence for our stage II colon cancer patients, using current clinico-pathological features. It is difficult at this juncture to determine why the number of lymph nodes retrieved per specimen has increased, but acknowledge that the pathologic techniques of lymph node retrieval have improved over the years.

In stage III colon cancer, the increasing number of positive lymph nodes present was a stronger indicator of risk, as expected. It has been shown that after adjusting for T stage, patients with N0 disease (0 positive lymph nodes) have an expected 5-year survival rate of 86%, compared to those with N2 disease (patients with >3 positive lymph nodes) with expected 5-year-survival rate of 69% [12]. Our results show that, after adjusting for adjuvant chemotherapy, each one-node increase in the number of positive lymph nodes was associated with a 24% increased risk of recurrence (HR 1.24; (118 – 1.31 95% CI) p < 0.001), verifying that an increasing number of positive lymph nodes is the most significant predictor of recurrence.

As expected, adjuvant chemotherapy for the stage III patients improved five-year disease-free survival rates, a finding consistent with those from the randomized clinical trials [2]. In a prognostic nomogram of all stages of colon cancer [13], adjuvant chemotherapy negated the negative prognostic factors of advanced T and N stage, and the c-index was 0.77 in predicting relapse for all stages of colon cancer. Although their reported c-index is promising, the model is driven by a larger proportion of stage I and IIA patients in the cohort and not by the stage III patients. Further, the published nomogram has not been validated by other institutions. The c-index of our multivariate model of the stage III colon cancer patients in our study was 0.70, much higher than the one found for stage II (c-index 0.56), however, not adequate. These findings illustrate the need to augment the TNM system for identifying individuals at high risk of recurrence.

A limitation of our study was the lack of follow-up of carcineoembryonic antigen (CEA). The role of CEA after surgical resection for colon cancer has been broadly assessed and, in spite of its widespread use, its utility has been controversial [14]. The argument in support of CEA in follow-up is based on the fact that early detection of asymptomatic recurrences is possible in patients with an elevated CEA. Opponents of CEA testing argue that approximately 40% of all colorectal recurrences do not demonstrate increased CEA levels [15], and no studies have demonstrated improved quality of life with frequent measurements. For these reasons, CEA measurements were not part of the surveillance for our patient cohort. Another limitation of our analysis was that all procedures took place in a specialized tertiary center, and the results may not be generalizable. In our institution, however, the fact that a group of specialized surgeons, medical oncologists and pathologists treated this patient cohort reduces the effects of treatment heterogeneity that exist in cohort studies of this nature. We are therefore better able to evaluate the independent predictiveness of current clinic-pathologic factors separately for stage II and III disease.

Further, in our study, we did not include any molecular markers of these tumors, including microsatellite instability (MSI) status. Our primary aim was to examine clinical and pathologic characteristics of stage II and III colon cancers, characteristics that are routinely obtained in community and specialty practice settings. Molecular profiling of colorectal tumors in the clinical setting carry great promise, but are not yet routinely performed as part of the current standard of care in the management of early stage colorectal cancers. For example, despite convincing evidence that MSI is a promising molecular marker with both prognostic and predictive value for chemosensitivity [16,17], it is not routinely obtained.

## Conclusions

In conclusion, colon cancer recurrence remains a considerable problem. The TN system, combined with all clinico-pathologic factors used today, fall short in predicting relapse, particularly for stage II disease. Identifying individual patients who might benefit from adjuvant chemotherapy, particularly for the stage II population is an unmet need. Integration of molecular characteristics of the tumors may lead to the development of a new staging system that will eventually surpass the current TNM system.

## Abbreviations

T: The penetration of tumor into the intestinal wall; N: The number of positive lymph nodes present; M: The presence of metastasis; QUASAR: Quick and simple and reliable study; SD: Standard deviation; CI: Confidence intervals; HR: Hazard ratio; AJCC: American Joint Commission on Cancer; SEER: Surveillance, Epidemiology, and End Results; CEA: Carcinoembryonic antigen; MSI: Microsatellite instability.

## Competing interests

None of the authors listed have any competing interests, neither financial, nor non financial. There was no funding for this study.

## Authors’ contributions

VT participated in the drafting of manuscript and developed study concept and design. DL performed critical revision of manuscript for intellectual content and database design. MH was the biostatistician and provided critical revision of manuscript for intellectual content. CL performed statistical analysis. PT participated in the drafting of the manuscript and development of study concept & design. All authors read and approve the final manuscript.

## Pre-publication history

The pre-publication history for this paper can be accessed here:

http://www.biomedcentral.com/1471-2407/14/336/prepub
